# Iron as an emerging therapeutic target in critically ill patients

**DOI:** 10.1186/s13054-023-04759-1

**Published:** 2023-12-04

**Authors:** Coralie Grange, François Lux, Thomas Brichart, Laurent David, Aymeric Couturier, David E. Leaf, Bernard Allaouchiche, Olivier Tillement

**Affiliations:** 1 MexBrain, 13 Avenue Albert Einstein, Villeurbanne, France; 2 Institut Lumière-Matière, UMR 5306, Université Claude Bernard Lyon1-CNRS, Villeurbanne Cedex, France; 3 Institut Universitaire de France (IUF), 75231 Paris, France; 4 Institut National des Sciences Appliquées, CNRS UMR 5223, Ingénierie des Matériaux Polymères, Univ Claude Bernard Lyon 1, Université Jean Monnet, 15 bd Latarjet, 69622 Villeurbanne, France; 5 Nephrology, American Hospital of Paris, Paris, France; 6 Division of Renal Medicine, Brigham and Women’s Hospital, Boston, MA USA; 7 Harvard Medical School, Boston, MA USA; 8 University of Lyon, University Lyon I Claude Bernard, APCSe VetAgro Sup UP, 2021. A10, Marcy L’Étoile, France

## Abstract

The multiple roles of iron in the body have been known for decades, particularly its involvement in iron overload diseases such as hemochromatosis. More recently, compelling evidence has emerged regarding the critical role of non-transferrin bound iron (NTBI), also known as catalytic iron, in the care of critically ill patients in intensive care units (ICUs). These trace amounts of iron constitute a small percentage of the serum iron, yet they are heavily implicated in the exacerbation of diseases, primarily by catalyzing the formation of reactive oxygen species, which promote oxidative stress. Additionally, catalytic iron activates macrophages and facilitates the growth of pathogens. This review aims to shed light on this underappreciated phenomenon and explore the various common sources of NTBI in ICU patients, which lead to transient iron dysregulation during acute phases of disease. Iron serves as the linchpin of a vicious cycle in many ICU pathologies that are often multifactorial. The clinical evidence showing its detrimental impact on patient outcomes will be outlined in the major ICU pathologies. Finally, different therapeutic strategies will be reviewed, including the targeting of proteins involved in iron metabolism, conventional chelation therapy, and the combination of renal replacement therapy with chelation therapy.

## Introduction

Iron plays a crucial role in numerous vital biological processes, such as oxygen transport, DNA synthesis, and ATP production [1]. The metabolism of iron is precisely regulated by several proteins that ensure its absorption, circulation, storage, and recycling. When iron homeostasis is disrupted, various pathologies can arise, including anemia due to iron deficiency and hemochromatosis resulting from iron overload [2]. While hemochromatosis is a well-known chronic condition characterized by excessive iron levels, acute dysregulation of iron homeostasis has also been linked to several pathologies, including sepsis [3], stroke [4], acute kidney injury (AKI) [5–7], and critical illness [8]. This review focuses on the connection between systemic iron excess, particularly the excess of non-transferrin bound iron (NTBI), and adverse clinical outcomes in critically ill patients. We discuss the multifactorial nature of NTBI in such patients and highlight that excess iron is a promising therapeutic target. Additionally, we explore potential therapeutic strategies to mitigate its detrimental effects.

## Iron: a double-edged sword

Iron is implicated in various crucial biochemical functions, such as oxygen transport and energy production, making it indispensable for life [2]. Interestingly, humans lack a specific pathway for excreting iron, so the regulation of iron homeostasis solely relies on proteins responsible for its absorption, transportation, storage, and recycling within the body [2, 9, 10]. After absorption, iron is released into the plasma and binds to transferrin, the primary iron transport protein in humans. Transferrin carries iron to target organs and tissues. The majority of this iron is directed to the bone marrow for erythropoiesis, with approximately 1.8 g of the total body iron content (approximately 3–4 g) contained within red blood cells (RBCs) [10, 11]. Macrophages phagocytize senescent RBCs, releasing intracellular iron for re-entry into the circulation [9]. This recycling process ensures a continuous flow of iron. Inside cells, iron is primarily stored in ferritin, which can accommodate up to 4,500 iron atoms in the form of a ferric oxide core [2, 12].

NTBI, also known as labile iron, free or loosely bound iron, or catalytic iron, has been identified in the bloodstream in various clinical scenarios. These terms refer to the same pool of iron that is not bound to transferrin or other iron-binding proteins, such as heme, ferritin, or hemosiderin [13]. For the sake of simplicity, this review will use the term NTBI to describe this labile iron pool, unless otherwise specified. NTBI is believed to encompass iron bound to albumin, citrate, acetate, amino acids, and other low-affinity ligands that have not been clearly defined [13, 14]. In healthy individuals, the transferrin saturation (TSAT) level is relatively low (~ 30–35%), which allows for the additional scavenging of NTBI released into the plasma. As a result, the levels of NTBI are often undetectable by most measurement methods [2, 13]. However, in pathological conditions associated with heavy iron overload, such as hemochromatosis, TSAT increases, and NTBI is frequently detected in the plasma within the range of 1–10 µM [15]. In comparison, the concentration of plasmatic iron, including both bound and free forms of iron, in a normal and healthy adult is approximately 20 µM [2]. Therefore, NTBI can account for up to 50% of the normal plasma iron.

NTBI level depends heavily on the quantification method used, which can vary widely due to the lack of a clinically available and universally accepted method. NTBI has been detected in the circulation of patients with primary hemochromatosis and beta-thalassemia, as well as during myeloablative therapy and stem cell transplantation [16, 17]. This increased pool of NTBI is highly toxic, as it generates deleterious reactive oxygen species (ROS), such as hydroxyl radicals (HO^•^), and promotes oxidative stress by catalyzing the Fenton and Haber–Weiss reactions [13]:

$$\begin{aligned} & {\text{Fe}}^{2 + } + {\text{H}}_{2} {\text{O}}_{2} \to {\text{Fe}}^{3 + } + {\text{HO}}^{ \cdot } + {\text{OH}}^{ - } \left( {\text{Fenton reaction}} \right) \\ & {\text{Fe}}^{3 + } + {\text{O}}_{2}^{ \cdot - } \to {\text{Fe}}^{2 + } + {\text{O}}_{2} \\ & {\text{O}}_{2}^{ \cdot - } + {\text{H}}_{2} {\text{O}}_{2} \to {\text{O}}_{2} + {\text{HO}}^{ \cdot } + {\text{OH}}^{ - } \left( {\text{Haber Weiss reaction}} \right) \\ \end{aligned}$$

Increased production of ROS can generate oxidative stress, which is characterized as an imbalance between oxidants and antioxidants in a biological system [18]. In particular, hydroxyl radicals are highly reactive and can lead to DNA degradation and lipid peroxidation [1]. Additionally, a newly described type of cell death called ferroptosis has emerged in the last decade firstly described by Stockwell et al*.* [19] in 2012 that is different from apoptosis, necrosis and autophagy and characterized by the accumulation of iron-mediated lipid-based ROS inside the cell [20, 21]. The iron-related mechanisms of ferroptosis are still not fully understood, but different studies have highlighted iron involvement and subsequent ROS formation, as increased cellular labile iron was observed during ferroptosis, whereas iron chelators were shown to prevent ferroptotic cell death [20].

Apart from its catalytic properties, NTBI may also indirectly lead to ROS production through its effects on macrophages that are phagocytic immune cells. Macrophages can mostly exist in two distinct phenotypes: (1) the classically activated or proinflammatory (M1 type) and (2) the alternatively activated or anti-inflammatory (M2 type) [22]. Iron influences macrophage polarization, most studies indicating polarization to the M1 proinflammatory phenotype in response to the presence of iron as recently reviewed by Ni et al [23] and Xia et al*.* [24]. Nevertheless, it is noteworthy that iron can also induce M2 activation in specific cases. Furthermore, iron accumulation in M1-polarized macrophages can lead to the persistent proinflammatory state observed in chronic and autoimmune diseases [25]. Ferroptosis can also activate macrophage recruitment due to the damage-associated molecular pattern molecules that are released by ferroptotic cells [22].

Finally, iron excess may be harmful by increasing the risk of infection [26]. Pathogenic bacterial development is strongly dependent on the presence of iron, as evidenced by their highly orchestrated mechanisms of acquiring iron, including heme uptake systems and secretion of siderophores to bind excess ferric iron [27]. High TSAT and the presence of plasma NTBI are associated with enhanced growth of siderophilic bacteria [28–30]. Iron availability is thus at the heart of the battle between infectious pathogens and host defenses [31]. To limit pathogen proliferation, the human organism has developed iron-withholding mechanisms called “nutritional immunity” to restrict iron availability from invading pathogens [32]. Under infectious conditions, hepcidin, the master hormonal regulator of systemic iron homeostasis, is overexpressed, resulting in a decrease in iron absorption by enterocytes and the sequestration of iron inside macrophages to reduce the extracellular iron concentration [33]. Dysregulation of iron homeostasis can thus easily tip the balance toward invading pathogens and against immune response.

**Fig. 1:**
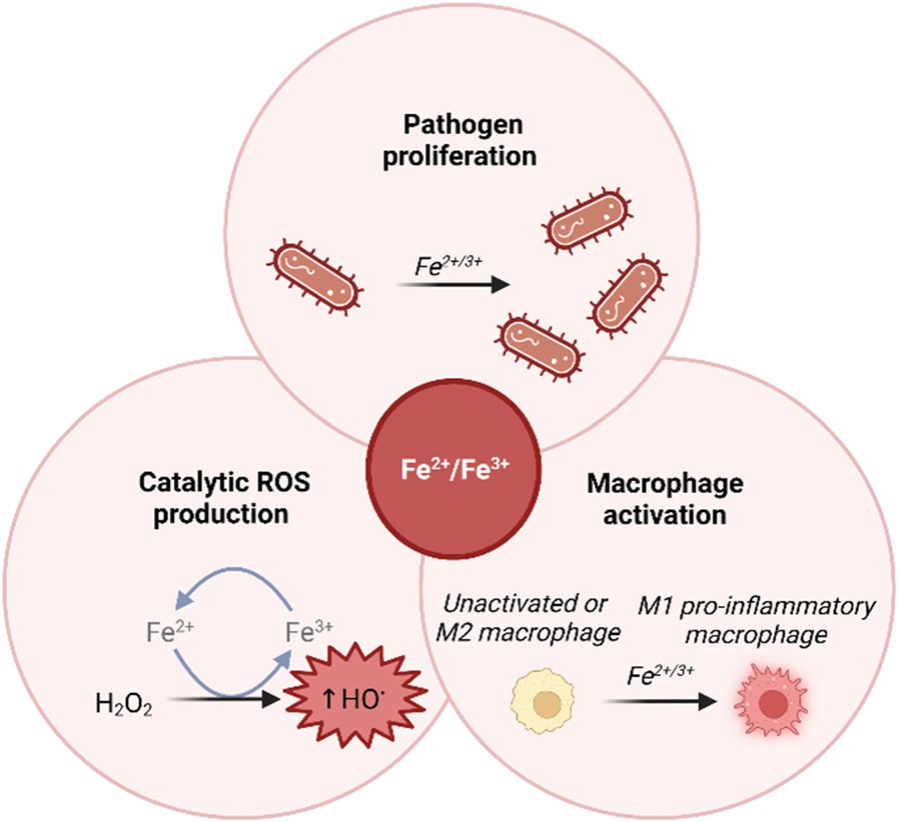
Detrimental effects of NTBI excess due to ROS production, macrophage M1 activation and its role in pathogen proliferation. H_2_O_2_: hydrogen peroxide, HO^•^: hydroxyl radical

In conclusion, NTBI, through its ability to catalyze the Fenton and Haber–Weiss reactions and thereby generate ROS, its macrophage activating effects, and its essential role in pathogen proliferation, may have toxic effects, induce oxidative stress, and cause severe cellular and tissue injury (Fig. 1).

## Iron metabolism in critically ill patients

Anemia is a common occurrence in critically ill patients, affecting up to 40% of those admitted to the ICU and leading to iron-deficient erythropoiesis [34, 35]. There are two primary types of anemia encountered in the ICU: iron deficiency anemia, characterized by total depletion of iron due to factors like repeated blood sampling or blood losses, and anemia of inflammation (AI), characterized by a functional iron deficiency marked by a reduction in serum iron levels alongside normal or elevated ferritin levels [34, 36]. In contrast to iron deficiency anemia, AI does not involve a reduction in iron stores but rather their preservation within iron-recycling macrophages [37, 38]: AI is better understood as a disorder of iron distribution rather than a true iron deficiency [39]. While the diminished availability of iron in AI may confer short-term benefits by establishing nutritional immunity against invading microbes, it also introduces risks such as the potential loss of homeostatic iron control and the risk of cellular iron excess [35]. Furthermore, anemia in ICU is typically addressed through blood transfusions, a practice that has been correlated with adverse clinical outcomes, including prolonged ICU length of stay and elevated mortality rates [40, 41]. Additionally, blood transfusions may lead to the release of free hemoglobin and free iron as a result of hemolysis [6]. On the other hand, the benefits of intravenous iron supplementation for anemia treatment in critically ill patients are still controversial [42, 43]. Hence, while a general positive correlation between anemia and iron content can be acknowledged, this relationship becomes more contentious when considering NBTI. Paradoxically, a lower iron content in plasma may be non-intuitively linked to a higher quantity of NBTI. A recent clinical trial (IRONMAN, ClinicalTrials.gov NCT02642562) has shown the interest of iron supplementation for patients with iron deficiency and chronic heart failure [44]. Other promising candidate to treat anemia is small molecule inhibitors of prolyl hydroxylase domain (PHD) enzymes that result in decreasing hepcidin levels and improving iron metabolism [45]. Despite an important prevalence of functional iron anemia in ICU, dysregulation of iron homeostasis leading to the release of NTBI has also been recently observed in critically ill patients.

The following review is focused on the association between iron excess and more particularly NTBI and the increased mortality in ICU.

## Sources of catalytic iron in critically ill patients

**Fig. 2:**
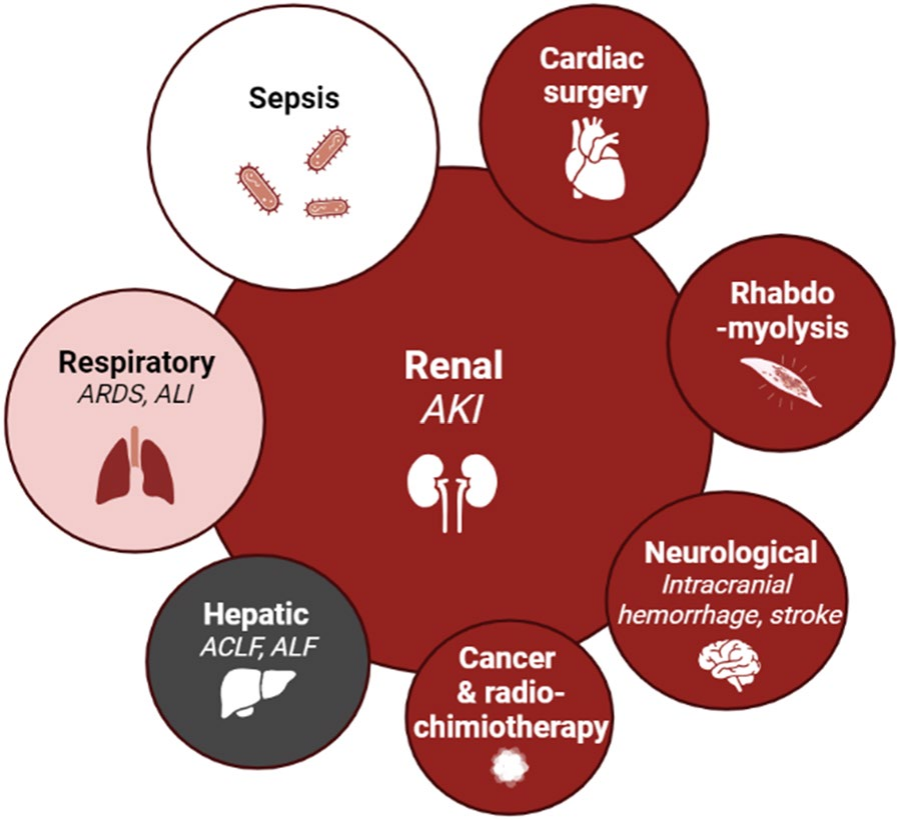
Nonexhaustive representation of the common etiologic factors for intensive care unit admissions each linked to confirmed or presumed NTBI liberation. Pathologies highlighted in black and dark red are linked to a sudden and substantial release of iron, often triggered by specific events that are associated with a significant amount of cell deaths. In respiratory pathologies (depicted in light red), iron accumulation in tissues may exacerbate the condition, contributing to a delayed release of iron. In the context of sepsis (illustrated in white), the scenario is less distinct. Although there is a hypothesis that NBTI is released with the occurrence of cell death, the intricate interplay between highly active microbes and macrophages further complicates the picture, as they avidly consume the liberated iron. *ARDS* acute respiratory distress syndrome, *ALI* acute lung injury, *ACLF* acute-on-chronic liver failure, *ALF* acute liver failure, *AKI* acute kidney injury

Due to the beneficial and toxic properties of iron, even a slight disruption in iron homeostasis can have significant clinical implications, particularly in critically ill patients [35]. Furthermore, this specific patient population frequently experiences acute organ injury, which can result in the release of NTBI into the bloodstream (Fig. 2).

### Hemolysis of red blood cells

Since RBCs contain most of the body’s iron, a probable source of iron release in critically ill patients is free hemoglobin. Consistent with this notion, several studies have observed a correlation between the plasma levels of free hemoglobin and NTBI [5, 7]. Hemolysis of RBCs releases free hemoglobin into the plasma, and the catabolism of hemoglobin leads to the further liberation of heme, bilirubin, and iron, which can overwhelm the relevant scavenging systems such as haptoglobin and hemopexin [46–48]. Van Avondt et al. [46] discussed the various mechanisms of hemolysis-induced AKI and emphasized the roles of NTBI, heme, and hemoglobin in mediating kidney damage through oxidative stress, inflammation, and nitric oxide depletion. Hemoglobin can be released by ex vivo hemolysis from blood transfusion and surgical procedures such as cardiopulmonary bypass, where extracorporeally circulated blood is exposed to nonphysiological surfaces and shear forces that may harm RBCs [6, 49–52]. Finally, in patients suffering from sepsis, free hemoglobin can be released due to blood transfusions, disseminated intravascular coagulation, and hemolytic pathogens [53].

### Rhabdomyolysis

In addition to its role for oxygen transport in circulating hemoglobin, iron has also a functional role in muscles, where it is bound to myoglobin, a hemoprotein responsible for oxygen transport and storage. In cases of severe muscle injury (rhabdomyolysis), much myoglobin (and consequently iron) can be released into the bloodstream [54]. Rhabdomyolysis is an acute syndrome characterized by skeletal muscle injuries resulting from trauma, inflammation, or ischemia, which leads to the release of breakdown products, including myoglobin [55]. AKI often occurs as a complication of rhabdomyolysis due to myoglobin-induced renal toxicity [54, 56]. Similar to free hemoglobin, free myoglobin can release heme, which can be further degraded by heme oxygenase into NTBI, leading to increased oxidative stress and tubular necrosis [56]. While the release of NTBI from heme has been proposed as the source of oxidative stress causing kidney injury, recent studies suggest that both myoglobin and heme themselves can cause lipid peroxidation and oxidative damage [57–59].

### Ischemia/reperfusion injury

Ischemia/reperfusion injury (IRI) may also result in an increase in NTBI levels, as demonstrated by numerous animal studies [60–62]. The exact causes of iron release into the circulation during IRI are not known, but iron might be released from ferritin either due to the acidic environment created by the ischemia or as a result of iron reduction by nicotinamide adenine dinucleotide phosphate (NADPH) or NADH, whose levels increase during ischemia [62–65]. This release of NTBI can induce oxidative stress and cellular damage in the kidneys, which can be aggravated during the reperfusion phase as the released NTBI is carried to the kidneys, introducing additional iron [48]. Interestingly, different studies have shown evidences of strong correlation between IRI and damages associated with ferroptosis [66].

### Liver cell death

The liver plays a crucial role in maintaining iron homeostasis by synthesizing most of the proteins involved in iron metabolism, such as hepcidin and transferrin, and serving as the primary storage site for iron [2]. Iron accumulation has been observed in the livers of patients with hemochromatosis and chronic liver disease [67, 68]. Again, the buildup of iron in the liver triggers the production of ROS, necrosis and apoptosis of hepatocytes, and inflammation [69]. It has been hypothesized that cell death in liver failure and necrosis are linked to the release of cellular content into the extracellular space [70, 71]. Several experts have suggested a direct role of iron in the progression from liver fibrosis to cirrhosis and multiorgan failure [69, 72]. In a study involving 20 patients with fulminant hepatic failure, catalytic iron was detected in 90% of them, with levels ranging from 0.2 to 6.2 µM, leading the authors to propose the damaged liver as a probable source of iron release [73].

### Cell death due to infection

Another source of NTBI in critically ill patients is the cell death that can occur in patients with infection, given the essential role of iron in the competition between infectious pathogens and host defense. Characterizing and measuring this iron release is challenging due to the complex situation where NTBI can rapidly be consumed by pathogens for their development as well as by macrophages fighting the same pathogens. Sepsis, a dysregulated host response to infection, is a major cause of AKI in the intensive care unit [3, 74]. As evocated previously, a common defense mechanism to limit iron availability to pathogens is increasing its intracellular storage, but this often reduces the serum iron level in septic patients and leads to anemia of inflammation [3]. Similarly, a dysregulation of iron homeostasis including anemia of inflammation has been observed in patients with COVID-19 [75]. Recently, ferroptosis has been identified as a potential cause of cell death in sepsis and cardiovascular damages in COVID-19 patients [76]. While iron accumulation in macrophages can promote pathogen destruction, ferroptosis can also lead to the death of macrophages and facilitate bacterial invasion [22, 77]. Furthermore, host defense mechanisms against infection involve various forms of cell death, including pyroptosis and necrosis, which can result in the release of intracellular contents into the extracellular medium [78, 79]. Pathogen infection leads to the death of many cell types, pathogen and immune cells alike, which may contribute to the release of NTBI.

## Higher plasma iron levels correlate with poor outcomes in the ICU

**Fig. 3:**
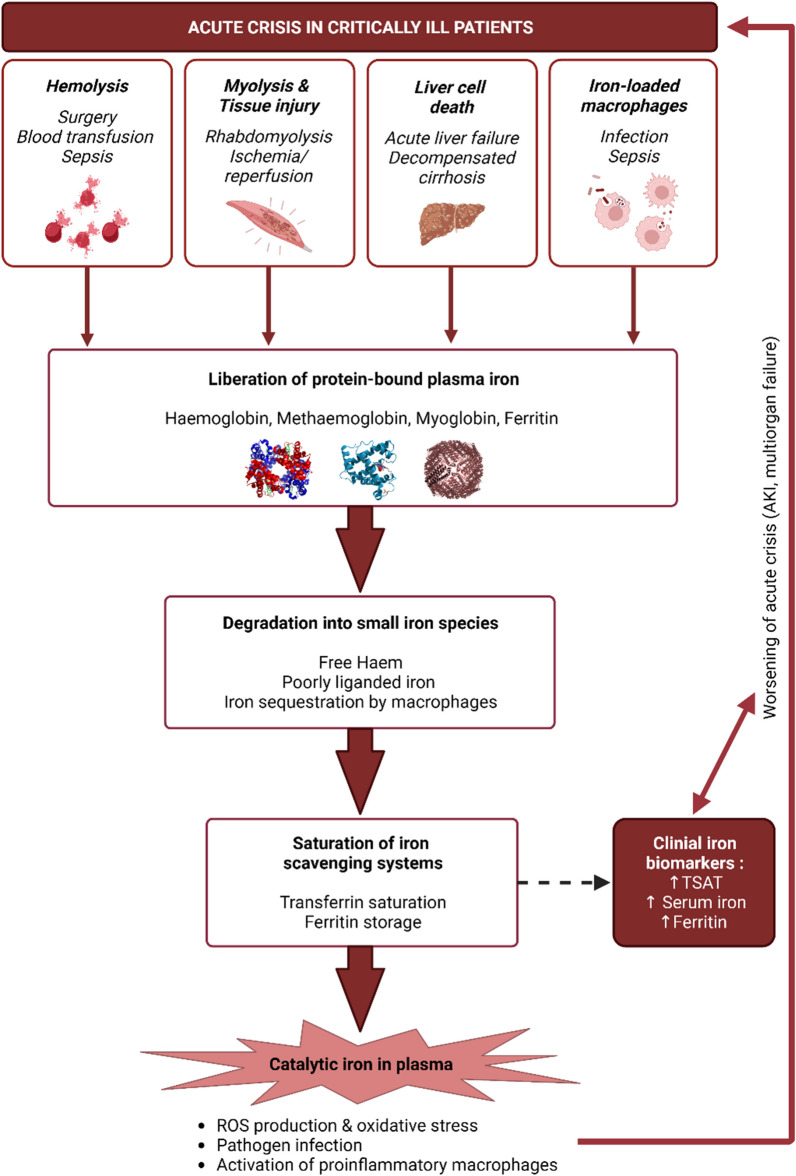
Catalytic iron release in the plasma of critically ill patients in acute crisis. Several acute crises (hemolysis, myolysis and tissue injury, liver cell death, and cell death in the case of infection) induce the release of iron-binding proteins and small iron species. This iron release is characterized by increased transferrin saturation and serum iron and ferritin levels, and it correlates with worsening clinical conditions, as catalytic iron can increase oxidative stress, promote pathogen infection, and activate proinflammatory macrophages

NTBI is released during acute crises observed in the ICU, leading to the saturation of physiological iron-scavenging systems and potentially exacerbating the acute crisis (Fig. 3). Monitoring iron parameters is therefore crucial in the ICU. Currently, total plasma iron and TSAT are commonly measured iron parameters to assess iron availability in the plasma (normal ranges in healthy patients are 20 µM and 30%, respectively) [2]. TSAT is calculated as the ratio of serum iron to serum total iron-binding capacity (TIBC), which can be determined from the transferrin concentration or directly measured by titration [80]. Ferritin, the iron storage protein, can also be found in low concentrations in the plasma (between 30 and 300 µg/L for men, between 15 and 150 µg/L for women) and reflects the iron stored in the body [9]. However, the iron content of ferritin can vary, and there is no accepted method for measuring ferritin-bound iron [81]. Furthermore, ferritin is an acute-phase protein whose synthesis is increased during inflammation by circulating cytokines such as IL-6, under which conditions serum ferritin may not correlate with the body's iron storage status [82]. Evaluating plasma iron availability in critically ill patients is complex, so we must measure multiple iron markers to better understand iron homeostasis.

Recently, Tacke et al. [8] conducted a study highlighting the dysregulation of iron homeostasis in critically ill patients admitted to the ICU with various pathologies. They observed that lower TSAT levels were associated with improved short- and long-term survival. Among the multifactorial pathologies observed in the ICU, AKI is a common syndrome affecting up to 60% of patients and is recognized as a significant cause of acute morbidity and mortality in critically ill patients [83–85]. In addition to drug-induced AKI, various pathological conditions, such as sepsis, cardiac surgery, or acute decompensated heart failure, can lead to AKI, which in turn can cause damage and dysfunction in other organs, leading to multiorgan failure [86]. The involvement of iron in AKI has been emphasized, and several reviews have discussed the role of NTBI and ferroptosis in the progression of AKI in animal models as well as in human AKI [48, 87–89].

Over the past 20 years, a growing body of evidence has emerged regarding the relationship between iron and mortality in the ICU. In a recent retrospective study involving 483 ICU patients with AKI, serum iron levels above 60 µg·dL^−1^ were associated with higher 28- and 90-day mortality (hazard ratio [HR]  1.83, 95% confidence interval (CI) 1.30–2.57 and [HR]  1.74, 95% CI 1.29–2.36, respectively) [90]. Similarly, elevated serum iron levels were found to be correlated with short- and long-term mortality in 1891 patients with sepsis. When stratified into quartiles based on serum iron levels, the 90-day mortality rate was significantly higher (*p* < 0.001) in higher quartiles of iron (26% in the first quartile vs. 36.2% in the fourth quartile) [91]. Dysregulation of iron metabolism was observed in a prospective study involving 61 septic patients, where higher TSAT was associated with reduced survival [92]. Likewise, a prospective study demonstrated dysregulation of iron metabolism in patients with acute-on-chronic liver failure (ACLF), with high TSAT being correlated with disease severity [93]. In a cohort of 292 patients with decompensated cirrhosis, alterations in iron metabolism were observed. Patients with ACLF had significantly higher TSAT (38% vs. 28%, *p* = 0.005) than those without ACLF. Furthermore, low transferrin concentration and high TSAT were associated with the severity of ACLF and increased short-term mortality [94]. Another retrospective study on patients with decompensated cirrhosis revealed a correlation between low transferrin concentration, high TSAT, disease severity, and short-term mortality [95]. These studies collectively demonstrate disrupted iron homeostasis in critically ill patients and an association between elevated serum iron levels or TSAT and poor prognosis in the ICU (Table 1).

**Table 1:** Correlations between iron parameters and adverse clinical outcomes in critically ill patients

Main pathology and cohort size	Iron parameters measured	Conclusion	Year
*Acute Kidney Injury*			
483 (AKI)	Serum iron Transferrin Ferritin	Serum iron higher than 60 µg·dL^−1^ (i.e., 10.7 µM) was associated with higher short- and long-term mortality	2020 [90]
121 (critically ill patients)	Plasma iron Transferrin and TSAT Ferritin Free hemoglobin Plasma catalytic iron	High plasma catalytic iron was correlated with a greater risk of AKI, RRT and mortality	2014 [7]
807 (AKI requiring RRT)	Plasma iron Transferrin and TSAT Ferritin Free hemoglobin Plasma catalytic iron	Higher plasma catalytic iron and lower hepcidin were associated with a greater risk of death	2019 [5]
*Sepsis*			
176 critically ill patients (57% with sepsis and 25% with septic shock)	Plasma catalytic iron	The severity of multiorgan dysfunction and the probability of death were associated with plasma catalytic iron and lipid peroxidation	2022 [96]
112 (septic) + 43 (nonseptic)	Serum iron Transferrin and TSAT Ferritin Hepcidin	Low iron (< 10.5 μM) and low TSAT (< 55%) were associated with short- and long-term survival	2016 [8]
61 (sepsis)	Serum iron Transferrin and TSAT Ferritin	Higher serum iron upon ICU admission, higher TSAT and lower transferrin were associated with higher mortality	2020 [92]
1891 (including 324 in septic shock)	Serum iron Transferrin Ferritin	Higher serum iron was independently associated with increased 90-day mortality	2018 [91]
*Liver disease*			
120 (ACLF) + 20 (DC) + 20 (healthy patients)	Serum iron Transferrin and TSAT Ferritin Hepcidin Cellular LIP	Iron metabolism and transport were dysregulated in ACLF patients, and more so in those with MOF. TSAT, hepcidin, and labile iron pool in macrophages correlate with disease severity	2015 [93]
292 (DC)	Serum iron Transferrin and TSAT Ferritin Hepcidin	Lower serum transferrin and higher TSAT correlated with poor short-term prognosis in patients with manifested organ failure	2017 [94]
286 (End-stage liver disease)	Transferrin Ferritin	High serum ferritin and lower serum transferrin were associated with greater 90-day mortality	2019 [97]
*Cardiac procedure*			
301 (cardiac surgery)	TSAT Ferritin Urine hepcidin	Higher TSAT and lower ferritin at 1 h of cardiopulmonary bypass were associated with the development of AKI	2019 [98]
14 (cardiac surgery)	Urine catalytic iron Urine NGAL	Evolution of increased urine catalytic iron and NGAL during cardiopulmonary bypass were correlated and may be indicative of early AKI	2013 [51]
250 (cardiac surgery)	Plasma iron Transferrin and TSAT Ferritin Free hemoglobin Plasma catalytic iron	Patients with the highest catalytic iron concentrations after operation had greater odds of AKI, hospital mortality, and postoperative myocardial injury	2015 [6]
806 (ACS and undergoing contrast exposure)	Catalytic iron	Catalytic iron was associated with increased mortality at 30 days. Contrast nephropathy was correlated with increased catalytic iron and mortality	2013 [99]
*COVID-19*			
120	Serum iron TSAT Ferritin Hepcidin Serum catalytic iron	Serum catalytic iron was positively associated with in-hospital mortality after adjusting for age and sex	2021 [100]
31	Serum iron TSAT Ferritin Trasnferrin	TSAT is extremely reduced at ICU admission and then significantly increases at days 3 to 6	2020 [101]
75 (associated with mucormycosis)	Serum iron Ferritin Hepcidin TIBC	Clinical severity of COVID-19-associated mucormycosis (CAM) was correlated with higher mortality and increased serum iron levels	2023 [102]

*ACLF* acute-on-chronic liver failure, *ACS* acute coronary syndrome, *AKI* acute kidney injury, *DC* decompensated cirrhosis, *ICU* intensive care unit, *LIP* labile iron pool, *MOF* multiorgan failure, *NGAL* neutrophil gelatinase-associated lipocalin, *RRT* renal replacement therapy, *TIBC* total iron-binding capacity, *TSAT* transferrin saturation

The PubMed and Web of Science databases were searched with a combination of the following terms and their synonyms: iron, correlation, mortality, intensive care unit, critically ill patients. In a second step, articles were searched for the main pathologies identified by combining the previous terms and acute kidney injury, cardiopulmonary bypass, liver failure, or sepsis

Several prospective studies have directly measured the concentration of NTBI in critically ill patients and correlated it with clinical outcomes. In a cohort of 121 ICU patients, higher plasma catalytic iron upon arrival to the ICU (measured using the bleomycin assay) was associated with an increased risk of AKI (odds ratio [OR] 1.67, 95% CI 1.04–2.67), renal replacement therapy (RRT) (OR 3.93, 95% CI 1.48–10.44), and hospital mortality (OR 2.93, 95% CI 1.52–5.63), even after adjusting for age, estimated glomerular filtration rate (eGFR) at enrollment, and the number of packed red blood cell transfusions within 48 h prior to ICU admission [7]. The same research team also highlighted that higher plasma concentrations of catalytic iron were significantly associated with an increased risk of death in AKI patients treated with RRT (approximately fourfold greater odds of death for patients in the highest quintile of catalytic iron concentrations compared to the lowest quintile) [5]. Cardiac surgery involving cardiopulmonary bypass is a well-known cause of AKI. A recent study suggested that an impaired capacity to manage NTBI release during cardiac surgery, as defined by lower concentrations of ferritin and hepcidin, as well as higher TSAT, were predictors of AKI. In a small cohort of patients undergoing cardiac surgery, urine catalytic iron and neutrophil gelatinase-associated lipocalin (NGAL), a predictor of AKI, were significantly increased [51]. Similarly, in a cohort of 250 patients undergoing cardiac surgery, Leaf et al. [6] found an elevated level of plasma catalytic iron at the end of cardiopulmonary bypass, which was associated with an increased risk of RRT, death, and other adverse outcomes, including AKI and myocardial injury. Specifically, on the first postoperative day, patients in the highest quartile of catalytic iron had a 3.99-fold-higher odds of AKI (*p* < 0.01) and a 6.71-fold-higher odds of RRT or in-hospital mortality (*p* = 0.02) than the lowest quartile [6]. A study involving 806 patients with acute coronary syndrome indicated that higher catalytic iron levels were associated with higher mortality [99]. Multiorgan failure is the leading cause of death in the ICU, and an association between plasma catalytic iron (measured using the bleomycin assay), the severity of multiorgan dysfunction, and the risk of death was established in a cohort of 176 critically ill patients [96]. In the last few years, with increased ICU admissions due to COVID-19, a study on 120 patients diagnosed with COVID-19 showed that serum catalytic iron levels were correlated with in-hospital mortality [100]. In summary, elevated levels of serum iron, TSAT, and particularly NTBI have each been associated with increased mortality and adverse outcomes in various multifactorial pathologies observed in the ICU (Table 1). Dysregulated iron homeostasis appears to be a common predictor of mortality in these studies, underscoring the need for therapeutic strategies to investigate whether neutralizing NTBI can improve clinical outcomes. Furthermore, the kidneys frequently bear the brunt of iron excess, and the presence of excessive NTBI in kidney failure may induce multiorgan failure, leading to a poor prognosis and high mortality [48, 86].

## Therapeutic strategies for the neutralization of catalytic iron in AKI

The overload of NTBI has emerged as a crucial therapeutic target to enhance clinical outcomes in critically ill patients, particularly in cases of AKI. Considering the diverse sources of iron release, several strategies have been investigated to counteract this pool of NTBI.

### Targeting iron metabolism proteins

Given the significant role of iron in the pathophysiology of AKI [87, 103], targeting iron metabolism may offer a viable therapeutic approach (e.g., by enhancing iron sequestration within ferritin). Ferritin possesses a substantial iron-scavenging capacity and has demonstrated a protective role in murine models of AKI [104]. Further investigation is required to determine its protective role against human AKI due to the limited and conflicting available data [87]. On the other hand, the administration of hepcidin has been considered a potential therapeutic target for the prevention and treatment of AKI in the ICU [105]. The use of exogenous hepcidin could restore iron homeostasis by increasing ferritin expression and iron sequestration, leading to renal protection and a reduction in renal oxidative stress and inflammation in animal models of AKI [106, 107]. Clinical trials investigating hepcidin agonists are underway, evaluating their potential use for various clinical indications [108].

Given the detrimental effects of both free hemoglobin and its degradation products (including heme and NTBI) on the kidney, one potential therapeutic strategy for treating hemolysis-induced or iron-mediated AKI is the administration of haptoglobin to enhance the scavenging of free hemoglobin [46]. A plasma-derived haptoglobin, available commercially in Japan, has been used to treat severe hemolysis during blood transfusions, extracorporeal circulation, and thermal injury, and limited evidence suggests that haptoglobin administration during cardiopulmonary bypass can reduce postoperative AKI [109, 110]. Similarly, hemopexin, the primary endogenous scavenger of free heme, could limit heme toxicity, but controversial results have been observed in animal models. Additionally, upregulation of heme oxygenase-1 (HO-1) has been proposed as a therapeutic strategy to protect against heme and iron-related nephrotoxicity. HO-1 is the enzyme responsible for breaking down heme into bilirubin, carbon monoxide, and ferrous iron, and it can also upregulate ferritin expression to facilitate the sequestration of released iron [88, 111]. A phase 2 clinical study demonstrated the safe induction of HO-1 in patients receiving renal transplants using heme arginate, but further large-scale studies are necessary to evaluate its protective effects on the kidneys [112]. These studies provide promising therapeutic avenues for protecting the kidneys against iron-mediated AKI, but additional research is required to confirm their potential efficacy.

### Iron chelation therapy

Chelation therapy was initially developed for the treatment of secondary hemochromatosis associated with hematological disorders and transfusion-induced iron overload [113]. Recently, Sharma and Leaf conducted a review of existing data on iron chelation for the prevention of AKI in both animal models and humans, highlighting the potential of iron-chelating agents as a new therapeutic strategy [103]. However, only a few clinical trials have evaluated the use of iron-chelating agents for AKI prevention (Table 2). There is an ongoing randomized, double-blind, placebo-controlled trial (DEFEAT-AKI, ClinicalTrials.gov NCT04633889) to assess whether prophylactic administration of intravenous deferoxamine (DFO) can reduce the incidence of AKI following cardiac surgery [114]. Another strategy in clinical trials is the combined administration of an antioxidant and an iron-chelating agent. A double-blind, randomized, placebo-controlled trial was conducted to evaluate the effect of N-acetylcysteine (NAC) plus DFO in patients with shock. A total of 80 critically ill patients with hypotension (defined as mean arterial blood pressure < 60 mmHg or the need for vasopressors) were included in the study. Administration of N-acetylcysteine (NAC) plus DFO did not decrease the incidence of AKI compared to placebo (65% vs. 67%, respectively), but it did reduce the severity of AKI, with 60% of patients in the placebo group experiencing stage 2 or 3 AKI compared to 37% in the NAC/DFO-treated group [115].

**Table 2:** Prior and ongoing clinical trials of iron-chelating agents for AKI prevention

Clinical setting	Iron chelation	Primary outcome	Results	NCT number	Year
CKD patients undergoing coronary angiography	Deferiprone: one immediate-release and two extended-release tablets (900 mg) 1–3 h prior to angiography, then every 12 h for 8 days	Biomarker evidence of AKI	Not published	NCT01146925 (completed)	2011 [116]
Critical ill patients who develop hypotension, (n = 30)	NAC bolus dose of 50 mg/kg/4 h, followed by 100 mg/kg/day for 2 days and DFO single dose (1 g) administered at 15 mg/kg/h	Serum levels of markers of oxidative damage and inflammatory response	NAC plus DFO administration decreased plasma markers of oxidative damage and creatinine level at hospital discharge	NCT00870883 (completed)	2012 [117]
Critical ill patients who develop hypotension, (n = 80)		Incidence of AKI	NAC plus DFO did not decrease the incidence of AKI but decreased the severity and duration of AKI	NCT00870883 (completed)	2016 [118]
Patients undergoing cardiac surgery	DFO 30 mg/kg by i.v. infusion over 12 h	AKI incidence	Not available	NCT04633889 (ongoing)	2022 [114]

*AKI* acute kidney injury, *CKD* chronic kidney disease, *DFO* Deferoxamine, *NAC* N-acetylcysteine

Iron-chelating agents show promise, but selecting the appropriate agent for the clinical setting is crucial. Three iron-chelating agents, namely DFO, deferiprone, and deferasirox, have been approved by the US Food and Drug Administration (FDA) for iron overload [113]. However, deferasirox is contraindicated in patients with chronic kidney disease due to its toxic effect on proximal tubular cells [103, 118]. As tubular necrosis is a common cause of AKI, deferasirox is likely not suitable for iron chelation in the ICU. Deferiprone, a second-line treatment for iron overload, is the most effective of the three chelating agents in removing cardiac iron due to its high lipophilicity. However, its short half-life (1.5–2.5 h), requiring three doses per day, and its oral mode of administration pose limitations for ICU patients [119]. Moreover, in infectious situations, clinical iron-chelating agents face limitations in terms of their affinity for ferric iron, which should be higher than that of bacterial siderophores, and the chelating agent should not be recognized by microbial siderophore transporters [120]. Notably, DFO is a natural siderophore, so it increases the risk of infection by certain pathogens [121]. For this reason, bacteremia is an exclusion criterion for the DEFEAT-AKI clinical trial mentioned earlier. Recently, a synthetic iron-chelating polymer called DIBI, with antimicrobial properties, has been developed [122]. A study comparing this chelating polymer with the three approved iron-chelating agents in two murine models of sepsis showed that DFO, deferiprone, or deferasirox did not significantly affect bacterial growth, unlike DIBI, which appeared promising for the treatment of sepsis due to its anti-inflammatory and antibacterial properties [123]. Hruby et al. [124] recently reviewed various iron-chelating polymers as a promising alternative to low-molecular-weight chelating agents in cases of iron overload. Different strategies can be employed to conjugate current chelating agents to polymers to improve their circulation time or reduce their side effects [124]. In conclusion, despite the proven therapeutic effects of common iron-chelating agents in iron overload diseases, these agents may not be well suited for the burst liberation of iron observed in the ICU, primarily due to their side effects. However, new iron-chelating agents, including chelating polymers, offer a promising alternative, but their efficacy needs to be confirmed through further animal studies and clinical trials.

### Combining RRT and iron chelation

**Fig. 4:**
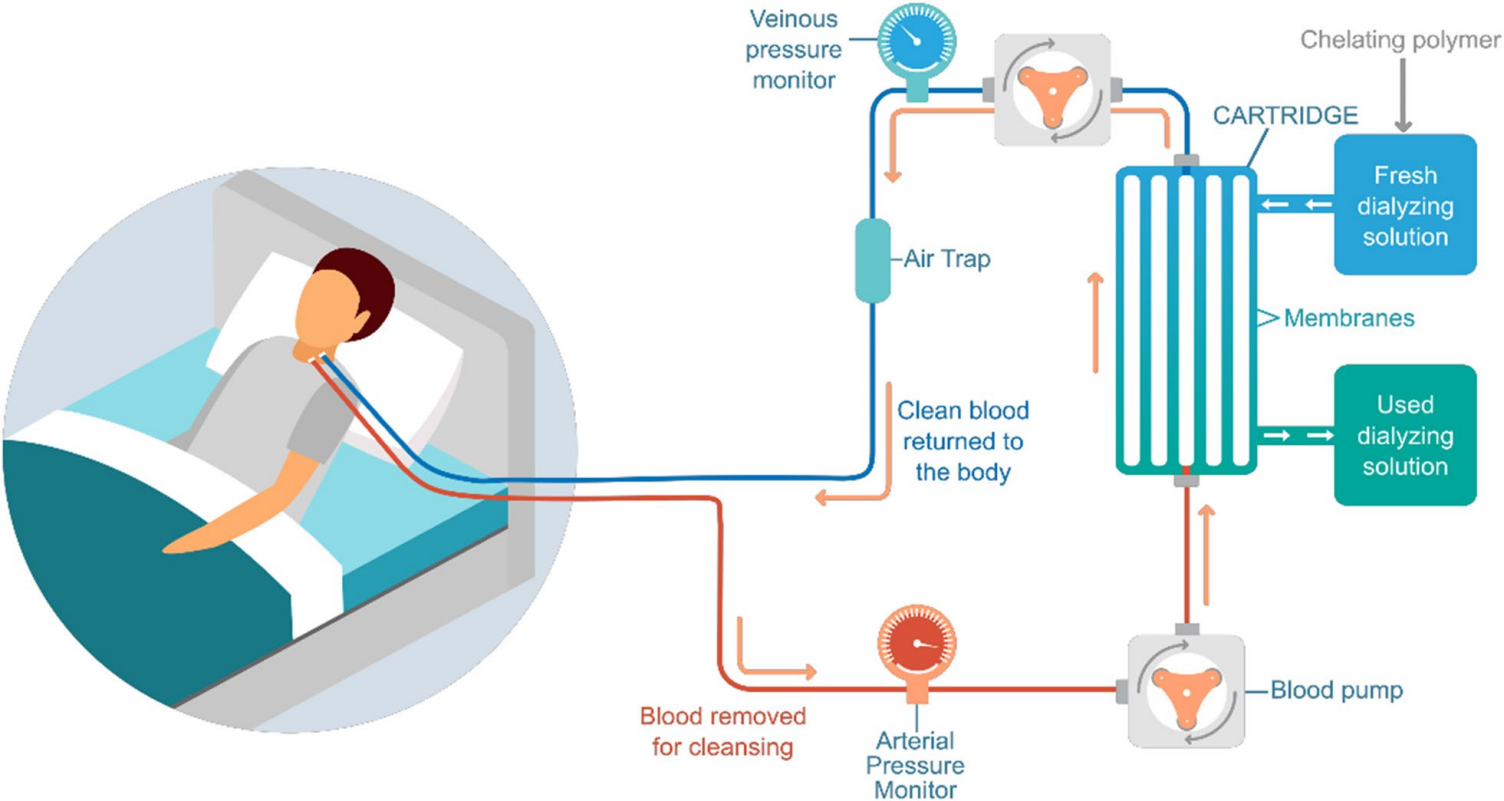
Schematic representation of metallic extraction using a chelating polymer in dialysate during hemodialysis

RRT is necessary for 5–10% of patients with AKI [125]. Its purpose is to support renal function by maintaining fluid and electrolyte balance and eliminating toxins through a semipermeable membrane, thereby preventing complications associated with AKI, such as volume overload and electrolyte and acid‒base imbalances [126–128]. Natuzzi et al. [129] and Grange et al. [130] proposed a potential solution for iron extraction in AKI patients requiring RRT involving the use of a chitosan-based chelating polymer in combination with hemodialysis (Fig. 4). By adding the chelating polymer to the dialysate, the diffusion equilibrium for iron is altered through metal complexation and the Fick diffusion law. This leads to enhanced extraction of metals while preventing their return to circulation, as the size of the polymer and the cutoff of the membrane pores hinder their passage. Implementing this approach in AKI patients undergoing hemodialysis could offer a noninvasive therapeutic option for iron extraction without requiring additional treatment or significant modification of the current standard of care. Clinical trials are necessary to validate the effectiveness of this medical device.

## Conclusion

Dysregulation of iron homeostasis has been observed in various pathologies commonly encountered in the ICU, particularly AKI, which is highly prevalent in this setting. The kidneys are often affected by catalytic iron-binding proteins released during acute crises, such as hemoglobin and myoglobin, as well as by excessive NTBI, which can induce oxidative stress and inflammation. Elevated iron parameters, particularly TSAT and NTBI, are correlated with unfavorable clinical outcomes in the ICU. Iron overload consistently emerges as a common predictor of in-hospital mortality in ICU patient studies, underscoring the urgent need for therapeutic interventions that neutralize this NTBI and the need to assess their impact on clinical outcomes. While classical iron-chelating agents could be considered potential therapeutic solutions, their suitability for ICU use is limited due to their side effect profile. Alternatively, direct targeting of proteins involved in iron metabolism holds promise as an alternative solution, but such approaches are still in the early stages of development. Lastly, addressing NTBI through the inclusion of iron chelation in commonly employed RRT protocols (via the addition of a chelating polymer to the dialysate) could provide a feasible therapeutic option for extracting NTBI and improving clinical outcomes in the ICU.

## Data Availability

Not applicable.
